# Drug Related Problems among Older Inpatients at a Tertiary Care Setting

**DOI:** 10.3390/jcm13061638

**Published:** 2024-03-13

**Authors:** Porrawee Pramotesiri, Krongtong Putthipokin, Sirasa Ruangritchankul

**Affiliations:** 1 Division of Geriatric Medicine, Department of Medicine, Faculty of Medicine, Ramathibodi Hospital, Mahidol University, Bangkok 10400, Thailand; porraweemd@gmail.com; 2 Clinical Pharmacy Unit, Pharmacy Division, Faculty of Medicine, Ramathibodi Hospital, Mahidol University, Bangkok 10400, Thailand; krongtong.putthipokin@gmail.com

**Keywords:** drug-related problems, older adults, adverse drug events, potentially inappropriate medications, drug–drug interactions

## Abstract

**Background:** Older persons are more likely to have multiple chronic diseases, leading to the simultaneous use of many medications. This situation results in increased drug-related problems (DRPs), which are the causes of adverse health outcomes. Therefore, we aimed to evaluate the prevalence of and associated risk factors for exposure to >1 criterion of DRPs among older adults admitted to a tertiary care hospital. **Methods:** We conducted a cross-sectional study involving 357 participants aged ≥60 years admitted to Ramathibodi Hospital from 1 February 2022 to 30 November 2022. The participants were evaluated for baseline characteristics, medications and DRPs and were classified into two groups, according to their exposure to DRPs: patients with exposure to ≤1 criteria and patients with exposure to >1 criterion of DRPs. Multivariate logistic regression analysis was performed to determine the independent risk factors for exposure to >1 criterion of DRPs. **Results:** Overall, 205 (57.4%) patients experienced >1 criterion of DRPs. Approximately 67.8%, 71.7% and 7.6% of the participants were exposed to at least one potentially inappropriate medication (PIM), drug–drug interaction (DDI) and adverse drug events (ADE), respectively. The most frequently prescribed PIMs were proton pump inhibitors (PPIs) (17.3%). Antineoplastics (48.1%) were the most frequently drug class related to ADEs. Overall, 37% of the ADEs in the current study were considered preventable ADEs. After adjustment for potential confounders, polypharmacy and the use of proton pump inhibitors, hypoglycemics, diuretics, psycholeptics, psychoanaleptics and cardiac therapy medications were correlated with a higher risk of exposure to > 1 criterion of PIMs, DDIs or ADEs. **Conclusions:** Therefore, comprehensive medication reviews and careful medication prescriptions are recommended in the geriatric population.

## 1. Introduction

Worldwide, aging populations have risen markedly during the past decade [1]. Similar to several other countries, Thailand became a “complete aged society” in 2022, with at least 20% of the population being ≥60 years of age [2]. The Thai geriatric population was almost 13.3 million in 2021 and is predicted to increase to 16.7 million by 2040 [2]. On account of a longer life expectancy, many older people tend to require more medical care and hospitalization. According to a preceding study, 65% of hospitalized patients were older persons, and they occupied 70% of the bed days [3]. In addition, older persons are more prone to multiple chronic conditions and diseases, including cardiovascular disease (CVD), diabetes mellitus (DM), hyperlipidemia (HLP), hypertension (HT), chronic obstructive pulmonary disease (COPD), anemia, chronic kidney disease (CKD) and cancer [4,5]. These adverse health problems result in an increased demand for simultaneous medication use. Multiple medication uses for acute medical problems pose a greater risk of drug-related problems (DRPs). A DRP is defined as the undesirable event involving medication therapy that interferes with desired medical outcomes [6]. Many classification systems have been developed to categorize DRPs, including the Helper–Strand classification [7], Cipolle’s classification [8] and the Pharmaceutical Care Network Europe (PCNE) [8]. Three common types of DRPs mentioned in clinical practice represent potentially inappropriate medications (PIMs), drug–drug interactions (DDIs), and adverse drug events (ADEs) [6,9,10,11,12,13]. Additionally, older populations are susceptible to DRPs, owing to age-related changes in pharmacokinetics and pharmacodynamics, cognitive decline and functional impairment [10,14,15]. DRPs increase the chance of unfavorable outcomes, such as poor quality of life, hospitalization, health care expenditure, morbidity and mortality [16,17,18,19,20]. As reported in previous studies, approximately 30% to 70% of hospitalized older patients developed DRPs [12,21,22,23]. Many studies have shown more than one DRP per person [24,25]. Furthermore, recent studies reported the prevalence of PIMs ranging from 28% to 73% [11,26,27,28], DDIs from 8% to 58% [12,29,30] and ADEs from 15% to 58% in the older population [13,31,32]. However, appropriate medicine management could prevent 15% to 70% of DRPs [33,34]. Therefore, healthcare providers should pay attention to comprehensive medication reviews and medication reconciliation to decrease the occurrence of DRPs [35,36,37]. 

Although many recent studies have explored DRPs in hospitalized patients, no study to date has reported the prevalence of and factors related to DRPs among hospitalized older patients in Thailand. The first aim of this study was to investigate the prevalence of and factors associated with exposure to >1 criterion of DRPs among hospitalized older patients in Thailand. The second aim was to explore the characteristics and prevalence of PIMs, DDIs and ADEs and describe the medications related to these DRPs.

## 2. Materials and Methods

### 2.1. Study Design, Setting and Participants

This cross-sectional study was conducted from 1 February 2022 to 30 November 2022. The current study involved 357 hospitalized older adults aged ≥60 years who were admitted to four internal medicine wards at Ramathibodi Hospital, Mahidol University, in the time period mentioned above. The sample size was calculated according to the prevalence of DRPs among older Thai adults (63.3%) in the previous study [38]. The participants were categorized into two groups: exposure to >1 criterion of DRPs or DRPs > 1 group and exposure to ≤1 criterion of DRP or DRP ≤ 1 group. The number of required participants was calculated from the following formula: N = Z_α_^2^pq/d^2^, where p represents the estimated prevalence with DRPs of 0.633, Z_α_ is the level of significance at the 95% confidence interval of 1.96, q is 1-p of 0.367 and d represents the precision of the estimate of 0.05. The calculated sample size for the study was 357. Patients with palliative care needs, hospital stays of <48 h and no response to the questionnaire or communication were excluded from the current study.

### 2.2. Data Collection and Measurement Tools

Data collection took place in the period between 1 February 2022 and 30 November 2022. Within 24 h of admission, medical staff reviewed and gathered the comprehensive data from the patients’ electronic medical records (EMRs), conducted face-to-face interviews and performed clinical assessments. A total of 357 participants completed the clinical assessment, and the questionnaire was administered through face-to-face interviews. All comprehensive data were gathered in their entirety during the study period. The data collected from the EMRs consisted of information on baseline characteristics, such as age, sex, race; body weight and height; health conditions: comorbidities and Charlson comorbidity index (CCI) scores [39]; psychological problems; current prescribed medications and documented DRPs; healthcare services: history of emergency room (ER) and outpatient department (OPD) visits, as well as hospital admission within the past 12 months; and laboratory results associated with exposure to >1 criterion of DRPs: white blood cell count, hemoglobin level, platelet count, partial thromboplastin time, prothrombin time, thrombin time, blood sugar level, sodium, potassium, calcium, magnesium, phosphorus, uric acid, blood urea nitrogen, creatinine, aspartate aminotransferase, alanine aminotransferase, alkaline phosphatase, serum albumin level, gamma-glutamyl transferase, total bilirubin, direct bilirubin, thyroid-stimulating hormone, free triiodothyronine and free thyroxine.

#### 2.2.1. Face-to-Face Interview-Administered Questionnaire

Within 24 h of admission, a questionnaire administered through a face-to-face interview was used to gather information on socio-economic variables (educational and marital status, income, medical insurance, career, history of caregivers and drug administrators, living situation and social activity), lifestyle behaviors (herbal use, and smoking and drinking status), cognitive function, depressive moods, activity of daily living (ADL) and clinical symptoms of DRPs. This questionnaire also evaluated geriatric syndromes [40], including falls, fecal and urinary incontinence, pressure ulcer, visual and auditory impairment, pain, weight loss, and immobility. Medical personnel assessed cognitive function using the Montreal Cognitive Assessment (MoCA). The total MoCA score ranges from 0 to 30 [41,42], with lower scores representing more severe decline in cognitive function, and a total score of <25 indicating cognitive impairment. In terms of mental health conditions, depression was evaluated using the Thai Geriatric Depression Scale-15 (TGDS-15) on a scale of 0 to 15, with a score >5 reflecting depression [43,44]. Functional ability was assessed using the Barthel ADL index [45] and the Lawton instrumental ADL (IADL) index [46,47]. The Barthel ADL index sums up the performance ability on the items of bathing, feeding, dressing, self-grooming, mobility, toilet use and incontinence. The index score has a range from 0 to 20, with lower scores reflecting a greater level of dependence. The Lawton IADL index assesses the performance of eight activities: shopping, handling finances, handling medications, food preparation, housework, laundry, telephone use and transportation. The total score ranges from a minimum of “0” to a maximum score of “8”, with lower scores indicating a higher level of functional incapacity. The data on socio-economics, lifestyle behaviors and geriatric syndrome were assessed by using a face-to-face interviewer-administered questionnaire. This questionnaire consists of binary questions, which are closed questions with only “Yes” or “No” answers; for example, “Have you had urinary incontinence in the geriatric condition section?” Participants were informed to answer “Yes” if he or she had urinary incontinence and “No” if he or she did not experience urinary incontinence. 

To assess the medication profiles, clinical pharmacists and geriatricians recorded the prescribed medications in the database. All medications were reported in terms of name, route of administration, frequency and dose. The medications were classified using the codes established by the Anatomical Therapeutic Chemical (ATC) classification system, which is the international standard system of medication usage and is recommended by the World Health Organization. Furthermore, the prevalence and characteristics of DRPs, namely, PIMs, DDIs and ADEs, were reported by clinical pharmacists and geriatricians. 

#### 2.2.2. Clinical Measurements

Medical practitioners assessed the participants’ body mass index (BMI) and clinical frailty within 24 h of admission as well. The BMI was defined as a person’s body weight in kilograms divided by the square of height in meters [48]. An experienced clinical physician evaluated the patients for frailty using the Clinical Frailty Scale (CFS), a clinical assessment tool for frailty in older populations [49]. The CFS score ranges from 1 (very fit) to 9 (terminally ill) [50]. 

#### 2.2.3. Definitions

- Polypharmacy (PP)

PP refers to the simultaneous use of five or more medications [51].

- Drug-related problem (DRP)

By definition, a DRP is any unpleasant event involving pharmacotherapy that interferes with achieving the desired goals of medical care [6]. The DRP is classified differently based on a specific focus. The classification systems established by Hepler and Strand [7], Cipolle and Morley [8] and the Pharmaceutical Care Network Europe (PCNE) [6] are widely used in research and clinical practice. In the current study, DRPs referred to only PIMs, DDIs and ADEs [52,53,54,55]. The outcome of interest was the exposure to DRPs, captured with the standard definition of this study and classified as a binary outcome: exposure to >1 criterion of DRPs and exposure to ≤1 criterion of DRPs.

- Potentially inappropriate medication (PIM)

PIM is defined as a medication that should be avoided because of an unfavorable risk ratio greater than the clinical benefit in the geriatric population [56,57]. In this study, PIMs were identified using the 2019 American Geriatrics Society (AGS) Beers Criteria^®^ (AGS Beers Criteria^®^), USA [58]. Furthermore, PIMs were evaluated and classified into five main categories: PIMs independent of individual’s medical condition, PIMs due to drug–disease and drug–syndrome interactions, drugs that must be used with caution in older adults, PIMs due to DDIs and drugs that must be adjusted in older adults with changes in kidney function [58].

- Drug–drug interaction (DDI)

The definition of DDI is the alteration of the activity of one drug by another drug, regardless of whether adverse events occur or not [59,60]. To determine DDIs, all drugs were evaluated using the Micromedex Drug Interaction Database [61]. Potential drug interactions were only taken into account for single pairwise drug combinations. All DDIs were reported with regard to severity and interaction effects. The severity of DDIs was categorized into four groups: minor, moderate, major and contraindicated DDIs [61].

- Adverse drug event (ADE)

An ADE is any detrimental experience resulting from medication exposure [62,63]. In this study, ADEs included adverse drug reactions, medication errors, drug abuse and drug overdose. The Naranjo algorithm [64] was used to determine the likelihood of adverse drug reactions occurring due to medication use. In addition, all participants were evaluated for the severity of ADEs by the Common Terminology Criteria for Adverse Events (CTCAE) [65] and were evaluated for ADE preventability by the Schumock and Thornton criteria [66].

### 2.3. Statistical Analysis

All statistical analyses in the current study were performed using the SPSS software package for Windows, version 25.0 (IBM Corp., Armonk, NY, USA). To describe baseline clinical characteristics and biochemical and medication profiles, continuous data are presented as mean ± standard deviation (SD) and median ± interquartile range (IQR), while categorical data are presented as percentage. In the comparison of all variables between patients with exposure to ≤1 criteria and >1 criterion of DRPs, Pearson’s chi-squared test or Fisher’s exact test was performed for categorical variables, and the unpaired Student’s *t*-test or Mann–Whitney U test was performed for continuous variables. Univariate logistic regression analysis was first used to identify potentially significant factors for exposure to >1 criterion of DRPs. A multivariate logistic model was then performed to adjust for significant variables, age, sex, Barthel ADL, Lawton IADL, CFS score, CCI score and cognitive function to explore independent risk factors for exposure to >1 criterion of DRPs. The results were reported as odds ratio (ORs) and 95% confidence intervals (CIs). A *p*-value of <0.05 was determined as statistical significance. 

### 2.4. Ethical Considerations

The research protocol was assessed and approved by the Committee on Human Rights Related to Research Involving Human Subjects, Faculty of Medicine Ramathibodi Hospital, Mahidol University (Protocol Number: COA. MURA2021/1068). All methods were carried out in accordance with the relevant guidelines and regulations in the Declaration of Helsinki. All participants provided written informed consent based on preference of literacy level before their participation. The copy of informed consent form provided to all participants was approved by the ethics committee. To protect confidentiality and privacy, patient data were coded and replaced with a participant identification number and date of birth. Furthermore, these personal confidential data were electronically stored in password-protected folders and used strictly by the approved research team for the purpose of this research. 

## 3. Results

### 3.1. Baseline and Clinical Characteristics, and Biochemical Parameters

In total, 357 older inpatients were enrolled during the study period. Of the 357 participants, 205 (57.4%) and 152 (42.6%) experienced >1 criterion and ≤1 criteria of DRPs, respectively. The ages of the participants ranged from 60 to 94 years, and the mean age of the total population was 71.9 (SD 2.7) years. Most of the participants were female (*n* = 194; 54.3%) and had received ≤12 years of education (*n* = 237; 66.4%). Less than 10% of the patients were single. The results of the descriptive and comparative analysis of baseline characteristics between the two groups are shown in Table 1. The number of hospitalized patients aged >70 years was significantly higher among patients with >1 criterion compared to those with ≤1 criteria of DRPs (*p* = 0.009). The BMI was not different between the two groups [23.6 (4.9) kg/m^2^ in the DRPs >1 group vs. 23.4 (3.9) kg/m^2^ in the DRP ≤1 group]. With regard to lifestyle patterns, 37.8%, 35%, and 27.5% of the total participants were alcohol drinkers, smokers and herbal medicine users, respectively. However, there was no significant difference in the prevalence of these behaviors between the two groups (*p* >0.05). Before admission, the number of participants living at a residential age care facility (RACF) was markedly higher among patients exposed to >1 criterion of DRP as compared to those exposed to ≤1 criteria of DRP (*p* = 0.001). With respect to discharge destination, the number of patients discharged to a RACF was significantly higher in the DRPs >1 group compared with those in the DRP ≤1 group (*p* = 0.002). In addition, a significant higher proportion of participants in the DRP ≤1 group were able to self-administer their medications compared to those in the DRPs >1 group (78.9% vs. 67.3%, respectively; *p* = 0.015). In terms of healthcare services, the number of hospitalized older patients with a history of ≥10 OPD visits or ≥5 doctor visits within 1 year was remarkably higher among patients with >1 criterion compared to those with ≤1 criteria of DRPs (*p* < 0.05). There was no substantial difference in the median length of hospital stay between the two groups (*p* = 0.846). 

In terms of comorbidities, patients with >1 criterion of DRPs had substantially more comorbidities and higher CCI scores than those with ≤1 criteria of DRP (*p* < 0.001), as shown in Table 2. In both groups, the three most common comorbidities were hypertension (85.4%), dyslipidemia (72.3%) and anemia (49.3%). Participants exposed to >1 criterion of DRPs were more likely to have HLP, HT, CKD, obstructive sleep apnea (OSA), anemia, arrhythmia, coronary artery disease (CAD) and congestive heart failure (CHF) than those with ≤1 criteria of DRP (*p* < 0.05). Additionally, CHF (9.5%), non-ST elevation, myocardial infarction (9.2%) and acute cholangitis (5.0%) were the three leading causes of hospital admission. 

Regarding geriatric syndromes and functional abilities, more than half of the total population had cognitive impairment (79.3%), as indicated by a MoCA score < 25, impaired IADLs (68.1%), sleep disorder (57.7%) and significant weight loss (53.8%) (Table 3). The participants with >1 DRPs tended to have greater frailty and dependence in IADL compared to those with ≤1 DRP (*p* < 0.001). Furthermore, the number of participants with a history of falls within the last 3 months or those ambulating with assistance were substantially higher in the DRPs >1 group than those in the other group (*p* < 0.05). Nevertheless, the number of participants with cognitive impairment (MoCA score < 25), depression (TGDS score >5) or impaired Barthel ADLs (Barthel ADLs score < 12) was not significantly different between the two groups. 

The mean prothrombin time and median blood urea nitrogen and creatinine levels were significantly higher in patients exposed to >1 criterion compared to those exposed to ≤1 criteria of DRPs (*p* < 0.05), as presented in Table 4. By contrast, the median alanine aminotransferase concentration, mean hemoglobin and free triiodothyronine concentration were markedly lower in patients with >1 criterion of DRPs compared to those with ≤1 criteria of DRPs. However, there was no apparent difference in the serum albumin, sodium, potassium, calcium, magnesium or phosphate levels between the two groups (*p* > 0.05). 

### 3.2. Medication Profiles

All prescribed medications classified by ATC codes are revealed in Table 5 and Table 6. The total number of prescribed medications in the current study was 3136, ranging from 1 to 23 per person. The median number of prescribed medications was 11 (IQR 8–13 medications) in patients who experienced >1 criterion of DRPs and 5 (IQR 3–8 medications) in those who experienced ≤1 criteria of DRPs. The number of participants exposed to PP was significantly higher among those with >1 criterion compared to those with ≤1 criteria of DRPs (97.1% vs. 59.9%, respectively; *p* < 0.001). In both groups, the three most commonly prescribed medications were lipid-modifying agents (62.2%), followed by vitamins (54.3%) and antithrombotic agents (46.2%). Among patients with >1 criterion of DRPs, the prescriptions of drugs for acid-related disorders, drugs used in diabetes, vitamins, mineral supplements, antithrombotic agents, cardiac therapy, antihypertensives, diuretics, beta-blocking agents, lipid-modifying agents, blood substitutes, calcium channel blockers, systemic hormonal preparations, immunostimulants, immunosuppressants, drugs for treatment of bone diseases, antiepileptics, psycholeptics, psychoanaleptics, antihistamines for systemic use and cough and cold preparations were substantially greater compared to those with ≤1 criteria of DRPs (*p* < 0.05). 

### 3.3. DRPs

The prevalence of DRPs is presented in Table 5 and Table 7. Of all 357 participants, 303 (84.9%) were exposed to at least 1 DRP of PIMs, DDIs and/or ADEs. Additionally, 98 (27.5%) patients were exposed to 1 DRP, 188 (52.7%) to 2 DRPs and 17 (4.8%) to a combination of PIMs, DDIs and ADEs. Approximately 67.8%, 71.7% and 7.6% of the total participants were exposed to at least 1 PIMs, DDIs and ADEs, respectively.

Of the 3136 prescribed medications, 612 PIMs were determined according to the 2019 AGS Beers Criteria^®^ in 357 participants, as shown in Table S1. The most frequently prescribed PIMs were proton pump inhibitors (PPIs) (17.3%) and benzodiazepines (13.8%). 

**Figure 1:**
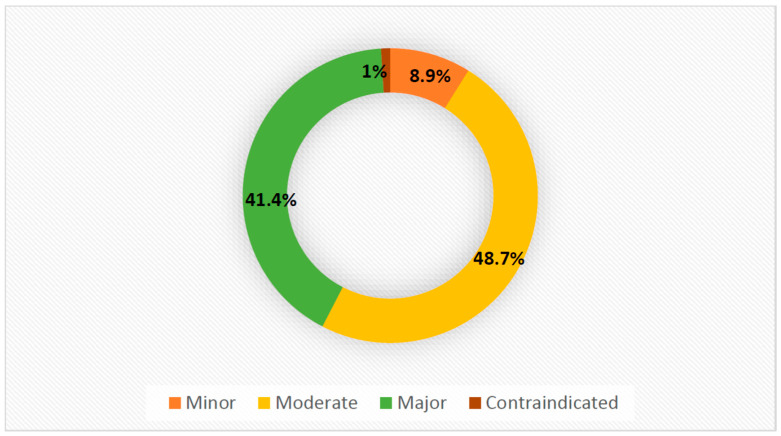
The severity of DDIs.

The severity and interactive effects of DDIs are reported in Figure 1 and Table S2. A total of 1210 medication combinations contributed to DDIs, as shown in Table 7. Approximately 50% of the total population was exposed to moderate DDIs. The medication combination often implicated in DDIs was amlodipine with simvastatin, the resulting interaction of which increases the risk of myopathy and rhabdomyolysis. However, these DDIs had no detrimental clinical outcomes. 

The ADEs and their preventability are shown in Table 7 and Tables S3–S6. Of the 27 reported ADEs, 15 (55.6%) and 12 (44.4%) were categorized as severe and life-threatening, respectively. More than 50% of the patients developed hematological problems (n = 19; 70.4%), including febrile neutropenia (*n* = 7; 25.9%). Antineoplastics (48.1%) and antithrombotic agents (22.2%) were the drugs most frequently related to ADEs. Overall, 37% of the ADEs in the current study were considered preventable ADEs. Anticoagulants, including warfarin, enoxaparin and dabigatran, accounted for 60% of the preventable ADEs, contributing to undesirable bleeding. 

### 3.4. Factors Associated with Exposure to >1 Criteria of DRPs 

According to the multivariate logistic regression analysis, significant variables were adjusted by age, sex, Barthel ADL, Lawton IADL, CFS score, CCI score and cognitive function (Table 8). After this adjustment, the factors associated with exposure to >1 criterion of DRPs were PP (OR 8.25, 95% CI 3.14–21.70) and increased use of medications for acid-related disorders (OR 3.82, 95% CI 2.08–7.00), drugs used in diabetes (OR 2.33, 95% CI 1.19–4.59), cardiac therapy (OR 3.19, 95% CI 1.29–7.89), diuretics (OR 8.20, 95% CI 3.11–21.65), psycholeptics (OR 4.59, 95% CI 1.80–11.69) and psychoanaleptics (OR 5.97, 95% CI 1.21–29.34). By contrast, age, sex, ADL, clinical frailty, comorbidities, a history of falls within the past 12 months and cognitive function were not associated with exposure to >1 criterion of DRPs.

## 4. Discussion

The present study revealed factors correlated with exposure to >1 criterion of DRPs and the characteristics of PIMs, DDIs and ADEs, as well as the medication related to these DRPs amongst hospitalized older patients aged ≥60 years during the study period, from 1 February 2022 to 30 November 2022. The prevalence of DRPs among older adults admitted to the medical department of our tertiary care hospital was 84.9%, which corresponds to the finding reported by Ramanath and Nedumballi [67]. Regarding the demographic characteristics, advanced age is a risk factor for DRPs due to its associated alterations in physiology, pharmacokinetics and pharmacodynamics, such as the deterioration of hepatic and kidney function. Furthermore, it has been found that older adults are more likely to be sensitive to the activities of certain drugs, such as anticholinergics and opioids, leading to DRPs [14,68]. Sluggett et al. [69] reported that older adults residing in RACF are liable to develop DRPs because of increased frailty and coexisting chronic diseases, resulting in the need for many drug therapy regimens and a risk of drug-related harm. The increase in OPD visits may also contribute to the rise in prescribed medications and DRPs [70,71]. 

In the current study, patients with high CCI scores were more prone to DRPs, which is consistent with the results of previous studies [9,72,73]. The most common chronic diseases were HT and HLP [74], whereas CHF was the most common cause of hospitalization in the geriatric population [9]. Similar to the results of preceding studies [75,76,77,78,79,80,81,82,83], hospitalized older persons exposed to many DRPs tended to have HLP, HT, CHF, CKD, OSA, CAD, arrhythmia and anemia. HT may contribute to multiple chronic diseases, including stroke, cognitive impairment, and CVD, which are treated with many medication combinations. This situation may potentially account for DRPs [84,85,86]. Additionally, many patients with CKD are vulnerable to DRPs due to their complex medication regimens and deterioration of drug excretion. Medication dosing mistakes often occur in patients with CKD. Therefore, the dose and dose interval should be adjusted to reduce inappropriate doses and possible toxicity. Pathophysiological changes related DRPs in patients with CHF include venous congestion, arterial hypoperfusion and neurohormonal activation, each of which may affect pharmacokinetics [87]. Hepatic congestion is a common feature in CHF patients and can progress to cirrhosis, leading to decreased drug metabolism and hepatic drug clearance [88]. Apart from hepatic dysfunction, kidney dysfunction is frequently found in CHF patients as a result of reduced renal blood flow and renal venous hypertension [89]. Furthermore, multiple complex medication regimens and several narrow therapeutic medications (e.g., digoxin and lidocaine) are used in CHF to balance water and sodium hemostasis and improve cardiac function [90], leading to a higher risk for DRPs [91,92,93].

With respect to the geriatric syndrome and psycho-cognitive status, we found that patients exposed to >1 criterion of DRPs tended to have more impaired ADL, frailty or a history of falls within the last 3 months than those who experienced ≤1 criteria of DRP, similar to findings from previous studies [94,95]. Frailty is a predictive factor of adverse clinical consequences, such as falls, fractures and functional inability [96]. The increasing number of DRPs in frail older adults may be explained through frailty-related pharmacokinetic and pharmacodynamic changes, such as reductions in the glomerular infiltration rate and hepatic drug clearance, especially in the phase II metabolism, which involves conjugation by coupling the medication to another molecule [97,98,99,100]. 

By contrast, we found no relationship between cognitive function and the increase in undesirable DRPs, which is in accordance with the findings reported by Paisansirikul et al. [38]. Unlike previous literature reviews, older adults with dementia have greater experience with DRPs, especially ADEs, owing to increased blood–brain permeability and sensitivity to the neurological effects of medications [101,102].

In terms of biochemical profiles, higher serum creatinine and lower hemoglobin levels are substantially associated with a greater incidence of DRPs [72,103,104]. The kidneys eliminate many types of prescribed drugs or their active metabolites. These renally excreted drugs should be prescribed based on the patient’s renal function or serum creatinine level to reduce exaggerated pharmacologic effects [105]. The common use of antithrombotic agents predisposes certain pharmacological therapies to develop ADEs, such as bleeding complications and anemia, especially in the older population [106]. The result of our study contrasts with those in the previous study, indicating that an increased serum albumin concentration is an important risk factor for potential DRPs [107,108]. Albumin accounts for 60% of drug-binding proteins. Medications binding to human serum albumin are usually pharmacologically inactive. On the contrary, the unbound portion of the medication could exert pharmacological activity [109]. Blood protein concentration plays a major role in the therapeutic dose and drug side effects. The free drug concentration is increased in hypoalbuminemia, leading to decreased therapeutic medication safety and increased risks of drug side effects and toxicity. 

PP is an outstanding factor in this study population, with individuals taking up to 23 medications per person. As expected, a high proportion of hospitalized older patients (81.2%) experienced PP. Similar to previous studies, most hospitalized older adults were exposed to PP [110,111,112,113].

The three most frequently prescribed medications were lipid-modifying agents, vitamins and antithrombotic agents. Antithrombotic agents are often prescribed to prevent and treat atherothrombotic events, CADs and atrial fibrillation (AF). Furthermore, new medications have been developed and launched, including non-vitamin K antagonist oral anticoagulants (NOACs) [114]. In the previous decade, the NOACs were approved by the Food and Drug Administration for the treatment of AF [115] and were recommended in clinical practice guidelines. With respect to vitamins, many hospitalized older patients develop malnutrition during hospitalization [116]. Hence, multiple macronutrients and micronutrients are required to maintain health status and diminish mortality. Thiamine and vitamin D deficiency are widely documented in the admitted geriatric population because of chronic diseases, age-related physiologic changes and nutritional difficulties [117,118]. Therefore, several vitamins have to be prescribed in clinical practice, based on patients’ individual characteristics and clinical requirements [119].

With regard to PIM use, most hospitalized older patients in the study (67.8%) were likely to experience at least 1 PIM, in agreement with studies by Silva et al. [120] and Varavithya et al. [121]. Nevertheless, the prevalence of PIMs varies according to healthcare settings, population structures and PIM assessment tools, including the AGS Beers Criteria^®^, the STOPP/START criteria, the PRISCUS list and the Laroche list [56,58,122,123]. In accordance with a recent study [120], the most common PIMs in older population involve medications for treatment diseases of the gastrointestinal (GI) and central nervous systems. According to the 2019 AGS Beers Criteria^®^, the most common medications implicated in PIMs are PPIs [120,121], followed by benzodiazepines [120]. The 2019 AGS Beers Criteria^®^ also indicate that older adults with long-term PPI use are susceptible to adverse events, such as *Clostridium difficile* infection, vitamin B12 deficiency, community-acquired pneumonia, fractures, and bone loss [58,124,125,126,127,128]. The inclusion of benzodiazepine as a PIM may be explained by the fact that benzodiazepines are well known to be associated with a risk of cognitive impairment, delirium, falls and fractures [58,129,130,131].

The results of the current study are consistent with those reported by Dagnew et al. [12], who found that the incidence of DDIs was high (71.1%) in the geriatric population. The majority of participants (47.9%) experienced one to five DDIs, and most DDIs (48.7%) were of moderate severity [12]. The most common therapeutic agents involved in DDIs were the combination of simvastatin and amlodipine. This drug interaction may contribute to the clinical risks of myopathy and rhabdomyolysis, similar to Dias et al.’s study, conducted in Brazil [132]. 

In two previous studies [133,134], approximately 30% of the overall population was exposed to ADEs, most of which were associated with antithrombotic and antineoplastic agents. Febrile neutropenia caused by antineoplastic agents was the most common ADE in our study. On the contrary, many studies have indicated an increased risk of GI-related ADEs, such as GI bleeding, in patients taking nonsteroidal anti-inflammatory drugs [135,136]. The ADEs observed in the study were categorized as severe and life-threatening in this susceptible population [34]. According to a previous study, older adults are more prone to severe adverse drug reactions because of a greater risk of polypharmacy and decline of hepatic function [137]. The prevalence of preventable ADEs in our study was 37% among all participants, which is lower than that reported in previous studies [34,133]. The prescribed medication category most often associated with preventable ADEs was anticoagulants (60%). This result contradicts that reported by Gray et al. [138], who found that half of the preventable ADEs resulted from psycholeptic agents.

Upon multivariate logistic regression analysis, PP, and the historical use of drugs for acid-related disorders, drugs used in diabetes, cardiac therapy, diuretics, psycholeptics, and psychoanaleptics were factors associated with exposure to >1 criterion of DRPs, which is consistent with the results of previous studies [103,107,108,139,140,141,142,143]. A high PP burden could be attributed to a significant experience of potential DRPs linked to increased DDIs and ADEs. Additionally, the number of taken medications is associated with the risk and severity of ADEs [144,145]. The toxicity of many drug combinations may be synergistic and greater than the total toxicity of medications used alone. Psycholeptics and psychoanaleptics contained in drugs classified as ATC code N (nervous system) are often considered “dirty” medications because they interact with many bonded molecular targets [146]. Hence, these medications may cause various ADEs [143]. Furthermore, several drugs classified as psycholeptics and psychoanaleptics are mentioned as PIMs according to the 2019 AGS Beers Criteria^®^ [58]. In addition, most psycholeptics and psychoanaleptics are metabolized through cytochrome P450, resulting in an increased risk of drug interactions [147,148]. Cardiac therapy drugs, classified as ATC code C01, comprise many medications with narrow therapeutic margins, such as digoxin and amiodarone, which usually interfere with other medications and come with potential drug interactions. Likewise, drugs used in diabetes, such as metformin and sulfonylurea, are also associated with DRPs. According to the 2019 AGS Beers Criteria^®^, long-acting sulfonylurea is notorious for increasing the risk of severe hypoglycemia [58]. Metformin may contribute to drug–disease interactions, which could raise the risk of lactic acidosis, particularly in patients with renal insufficiency [58,131,149]. Diuretics can affect age-related homeostasis, resulting in a greater risk of orthostatic hypotension, falls and electrolyte imbalance [149]. On the other hand, age, sex, ADL, clinical frailty, comorbidities, a history of falls within the past 12 months and cognitive function were not correlated with exposure to >1 criterion of DRPs, which is in contrast with preceding studies [38,150,151,152,153]. 

To the best of our knowledge, this is the first study to demonstrate the prevalence of and factors associated with exposure to >1 criterion of DRPs among hospitalized older patients in Thailand. Furthermore, this study revealed the characteristics of PIMs, DDIs and ADEs and their related medications. The main strength of the present study is the involvement of medical assessors, who conducted many clinical measurements and gathered comprehensive health data from several domains using a combination of EMR reviews and face-to-face interviews. Additionally, physicians and clinical pharmacists evaluated all DRPs and related medications using various standard assessment tools.

However, several limitations of this study should be mentioned. First, the study focused on older adults aged ≥60 years admitted to a tertiary care hospital. In addition, many older inpatients, such as those with palliative care needs, hospital stays <48 h and no response to the questionnaire or communication were excluded from the current study. Therefore, the results may need to be more generalizable. Second, the cross-sectional nature of this study restricts the determination of a temporal relationship between associations and outcomes. Third, patients with mild dementia who were able to communicate were still included in the study. This might have contributed to inaccurate information retrieval and recall bias. Fourth, the absence of the exact etiology and mechanism of ADEs may have led to difficulty in preventing ADEs. Finally, only DDIs involving a single pairwise medication combination were considered in the current study, but we did not include drug interactions from combinations of three or more medications. 

The essential results, including independent risk factors, could be included into a comprehensive measurement tool and geriatric assessment to identify DRPs in high-risk older individuals. Early detection and prevention of DRPs could be achieved through appropriate medication management. Additional prospective cohort studies are needed to assess adverse outcomes of DRPs, such as readmission, revisit to ER and mortality. Further cohort studies would encourage healthcare personnel to be aware of DRPs and their detrimental outcomes and to promote medication management, such as comprehensive medication reviews and medication reconciliations in order to optimize the prescription of medication for this susceptible population. In addition, an electronic prescribing system, which is a computer-based system used to create drug prescriptions and monitor and report drug-related problems, has long been implemented in western countries [154]. The prescribing system could prevent medication errors, prescribing abuse, drug interaction and adverse drug reactions [155,156]. Therefore, the use of this computer-based system should be widely encouraged in clinical practice to alert physicians and help them avoid DRPs. Additionally, an academic training program regarding appropriate medication use should be provided to physicians and clinical pharmacists. These medication management efforts would address potential DRPs and diminish their adverse consequences. 

## 5. Conclusions

This study confirms that a high proportion of hospitalized older patients are susceptible to PIMs, DDIs and ADEs. The independent factors associated with exposure to >1 criterion of DRPs were PP and the use of medications for acid-related disorders, drugs used in diabetes, cardiac therapy, diuretics, psycholeptics and psychoanaleptics. Therefore, the findings from this study should be utilized to develop comprehensive assessment tools for the early detection and prevention of DRPs in this high-risk population. Furthermore, medical staff should be encouraged to perform comprehensive medication reviews to address preventable DRPs. 

## Figures and Tables

**Table 1:** Baseline characteristics among hospitalized older patients classified by the exposure to DRPs.

Characteristics	All (n= 357)	DRPs > 1 (n = 205)	DRP ≤ 1 (n = 152)	*p*-Value
		N (%)	N (%)	
Age in years, mean (SD) ^a^	71.9 (SD 2.7)	72.7 (SD 7.8)	70.9 (SD 8.1)	0.037 ^#^
≤70 years	164 (45.9)	82 (40.0)	82 (53.9)	0.009 *
>70 years	193 (54.1)	123 (60.0)	70 (46.1)	
Female	194 (54.3)	120 (58.5)	74 (48.7)	0.065 *
Male	163 (45.7)	85 (41.5)	78 (51.3)	
**Educational status**				
≤12 years	237 (66.4)	138 (67.3)	99 (65.1)	0.666 *
>12 years	120 (33.6)	67 (32.7)	53 (34.9)	
**Marital status**				
Single	23 (6.4)	14 (6.8)	9 (5.9)	0.098 *
Married, widowed, or separated	334 (93.6)	191 (93.2)	143 (94.1)	
**Anthropometry**				
Body weight (kg), mean (SD) ^a^	59.5 (SD 12.6)	58.9 (SD 13.1)	60.2 (SD 12.0)	0.347 ^#^
Body mass index (kg/m^2^), mean (SD) ^a^	23.5 (SD 4.6)	23.6 (SD 4.9)	23.4 (SD 3.9)	0.716 ^#^
**Lifestyle**				
Drinking	135 (37.8)	75 (36.6)	60 (39.5)	0.578 *
Smoking	125 (35.0)	70 (34.1)	55 (36.2)	0.690 *
Herbal medicine use	98 (27.5)	56 (27.3)	42 (27.6)	0.948 *
**Socioeconomic status**				
Living alone	51 (14.3)	31 (15.1)	20 (13.2)	0.600 *
Living at residential age care facility	77 (21.6)	57 (27.8)	20 (13.2)	0.001 *
Discharge to residential age care facility	103 (28.9)	72 (35.1)	31 (20.4)	0.002 *
No caregiver	236 (66.1)	124 (60.5)	112 (73.7)	0.009 *
Self-drug administration	258 (72.3)	138 (67.3)	120 (78.9)	0.015 *
**Healthcare services**				
Paying for health care; self-paid	293 (82.1)	171 (83.4)	122 (80.3)	0.443 *
History of ≥10 OPD visits within 1 year	239 (66.9)	153 (74.6)	86 (56.6)	<0.001 *
History of >1 hospital admission within 1 year	44 (12.3)	31 (15.1)	13 (8.6)	0.062 *
History of >1 ER visit within 1 year	44 (12.3)	27 (13.2)	17 (11.2)	0.572 *
Number of doctors ≥5 persons	193 (54.1)	127 (62.0)	66 (43.4)	0.001 *
LOS, median (IQR) ^b^	6 (IQR 3.5–10)	6 (IQR 3–10)	6 (IQR 4–10)	0.846 ^+^

Data are presented as mean (standard deviation), n (%) or median (interquartile range). * Chi-square test, ^#^ Student’s *t*-test, ^+^ Mann–Whitney U test. ^a^ Variable represented as mean (SD). ^b^ Variable represented as median (IQR). **Abbreviations:** DRP—drug-related problem; SD—standard deviation; IQR—interquartile range; kg—kilogram; m—meter; ER—emergency room; OPD—outpatient department; LOS—length of stay.

**Table 2:** Clinical characteristics among hospitalized older patients classified by their exposure to DRPs.

Characteristics	All (n = 357)	DRPs > 1 (n = 205)	DRP ≤ 1 (n = 152)	*p*-Value
		N (%)	N (%)	
**Comorbidities**				
No. of chronic diseases, median (IQR) ^a^	5 (IQR 3–6)	5 (IQR 4–7)	4 (IQR 3–5)	<0.001 ^+^
CCI, median (IQR) ^a^	6 (IQR 4–8)	6 (IQR 5–8)	5 (IQR 3–7)	<0.001 ^+^
CCI score ≥5	257 (72.0)	166 (81.0)	91 (59.9)	<0.001 *
Dyslipidemia	258 (72.3)	158 (77.1)	100 (65.8)	0.019 *
Coronary artery disease	104 (29.1)	76 (37.1)	28 (18.4)	<0.001 *
Congestive heart failure	83 (23.2)	65 (31.7)	18 (11.8)	<0.001 *
Arrythmia	96 (26.9)	70 (34.1)	26 (17.1)	<0.001 *
Hypertension	305 (85.4)	191 (93.2)	114 (75.0)	<0.001 *
Cirrhosis	26 (7.3)	14 (6.8)	12 (7.9)	0.702 *
Osteoporosis	17 (4.8)	12 (5.9)	5 (29.4)	0.261 *
Osteoarthritis	9 (2.5)	5 (2.4)	4 (2.6)	0.909 *
Diabetes mellitus	161 (45.1)	101 (49.3)	60 (39.5)	0.066 *
Chronic kidney disease	150 (42.0)	106 (51.7)	44 (28.9)	<0.001 *
Anemia	176 (49.3)	111 (54.1)	65 (42.8)	0.033 *
Depression	13 (3.6)	10 (4.9)	3 (2.0)	0.147 *
Dementia	5 (1.4)	5 (2.4)	0 (0.0)	0.075 *
Cerebrovascular disease	59 (16,5)	38 (18.5)	21 (13.8)	0.235 *
Cataract	72 (20.2)	44 (21.5)	28 (18.4)	0.479 *
Malignancy	70 (19.6)	45 (22.0)	25 (16.4)	0.195 *
Obstructive sleep apnea	14 (3.9)	12 (5.9)	2 (1.3)	0.029 *
**Primary diagnosis**				
Congestive heart failure	34 (9.5)	26 (12.7)	8 (5.3)	0.018 *
Acute cholangitis	18 (5.0)	9 (4.4)	9 (5.9)	0.513 *
NSTEMI	33 (9.2)	20 (9.8)	13 (8.6)	0.698 *

Data are presented as mean (standard deviation), n (%), or median (interquartile range). * Chi-square test, ^+^ Mann–Whitney U test. ^a^ Variable represented as median (IQR). **Abbreviations:** DRP—drug-related problem; CCI—Charlson comorbidity index; IQR—interquartile range; NSTEMI—non-ST elevation myocardial infarction.

**Table 3:** Geriatric conditions among hospitalized older patients classified by their exposure to DRPs.

Characteristics	All (n= 357)	DRPs > 1 (n = 205)	DRP ≤ 1 (n = 152)	*p*-Value
		N (%)	N (%)	
**Geriatric conditions**				
MoCA score, mean (SD) ^a^	17.5 (SD 7.0)	17.3 (SD 6.9)	17.8 (SD 7.3)	0.486 ^#^
MoCA score < 25	283 (79.3)	168 (82.0)	115 (75.7)	0.147 *
TGDS score, median (IQR) ^b^	3 (IQR 2–4.5)	3 (IQR 2–5)	2 (IQR 1–4)	0.088 ^+^
TGDS score > 5	66 (18.5)	39 (19.0)	27 (17.8)	0.761 *
BADLs, mean (SD) ^a^	18.0 (SD 3.8)	17.8 (SD 3.8)	18.4 (SD 3.8)	0.734 ^#^
BADL impairment	33 (9.2)	20 (9.8)	13 (8.6)	0.162 *
IADLs, median (IQR) ^b^	6 (IQR 3–8)	5 (IQR 2–7.5)	7 (IQR 4–8)	<0.001 ^+^
IADL impairment	243 (68.1)	154 (75.1)	89 (58.6)	0.001 *
CFS score, median (IQR) ^b^	4 (IQR 3–5)	4 (IQR 3–5)	3 (IQR 3–4)	<0.001^+^
CFS score ≥5	107 (30.0)	72 (35.1)	35 (23.0)	0.014 *
Sleep disorder	206 (57.7)	127 (62.0)	79 (52.0)	0.059 *
Urinary incontinence	114 (31.9)	71 (34.6)	43 (28.3)	0.204 *
Fecal incontinence	61 (17.1)	39 (19.0)	22 (14.5)	0.259 *
History of fall within the past 3 months	106 (29.7)	74 (36.1)	32 (21.1)	0.002 *
History of delirium	34 (9.5)	16 (7.8)	18 (11.8)	0.199 *
Pressure ulcer	10 (2.8)	8 (3.9)	2 (1.3)	0.199 *
Visual impairment	37 (10.4)	21 (10.2)	16 (10.5)	0.931 *
Hearing impairment	1 (0.3)	1 (0.5)	0 (0.0)	1.000 *
Significant weight loss	192 (53.8)	112 (54.6)	80 (52.6)	0.707 *
Artificial enteral nutrition	12 (3.4)	7 (3.4)	5 (3.3)	0.948 *
Pain	116 (32.5)	60 (29.3)	56 (36.8)	0.131 *
Ambulation with assistance	120 (33.6)	81 (39.5)	39 (25.7)	0.006 *

Data are presented as mean (standard deviation), n (%), or median (interquartile range). * Chi-square test, ^#^ Student’s *t*-test, ^+^ Mann–Whitney U test. ^a^ Variable represented as mean (SD). ^b^ Variable represented as median (IQR). **Abbreviations:** SD—standard deviation; IQR—interquartile range; MoCA—Montreal Cognitive Assessment; TGDS—Thai Geriatric Depression Scale; BADLs—basic activities of daily living; IADLs—instrumental activities of daily living; CFS—Clinical Frailty Scale; DRP—drug-related problem.

**Table 4:** Laboratory results among hospitalized older patients classified by their exposure to DRPs.

Characteristics	All (n = 357)	DRPs > 1 (n = 205)	DRP ≤ 1 (n = 152)	*p*-Value
White blood cell count (cells/mm^3^), median (IQR)	7540 (5405–9720)	7560 (5890–9975)	7170 (5247–8880)	0.351 ^+^
Hemoglobin (g/dL), mean (SD)	10.6 (2.1)	10.4 (1.9)	10.9 (2.2)	0.046 ^#^
Platelet (cell/mm^3^), median (IQR)	232,000 (159,500–297,500)	224,000 (151,000–293,500)	250,000 (205,000–292,000)	0.257 ^+^
Blood urea nitrogen (mg/dL), median (IQR)	18 (12–29)	25.0 (16.0–36.5)	14.0 (10.0–24.8)	<0.001^+^
Creatinine (mg/dL), median (IQR)	1.1 (0.8–1.7)	1.2 (0.8–2.1)	0.8 (0.6–1.1)	<0.001^+^
Sodium (mmol/L), mean (SD)	136.9 (3.7)	136.7 (3.7)	137.2 (3.8)	0.248 ^#^
Potassium (mmol/L), mean (SD)	4.1 (0.5)	4.1 (0.5)	4.1 (0.5)	0.742 ^#^
Calcium (mg/dL), mean (SD)	8.8 (0.8)	8.8 (0.8)	8.7 (0.8)	0.156 ^#^
Magnesium (mg/dL), mean (SD)	1.9 (0.3)	1.9 (0.3)	1.9 (0.3)	0.930 ^#^
Phosphate (mg/dL), mean (SD)	3.5 (1.2)	3.6 (1.2)	3.5 (1.1)	0.297 ^#^
Aspartate aminotransferase (U/L), median (IQR)	34 (23.5–53)	37 (24–49)	35.5 (25.3–68.3)	0.256 ^+^
Alanine aminotransferase (U/L), median (IQR)	24 (14–48)	23 (12–45.5)	25.5 (16–58)	0.031 ^+^
Alkaline phosphatase (U/L), median (IQR)	91.5 (69,3–126.0)	90 (66–122.5)	93 (76–109.8)	0.950 ^+^
Total bilirubin (mg/dL), median (IQR)	0.7 (0.5–1.0)	0.6 (0.5–1.1)	0.7 (0.5–1.2)	0.309 ^+^
Albumin (g/L), mean (SD)	30.4 (6.5)	30.7 (6.1)	30.0 (6.8)	0.340 ^#^
Blood sugar (mg/dL), mean (SD)	132.5 (45.1)	135.7 (46.8)	128.2 (42.7)	0.121 ^#^
Thyroid stimulating hormone (uIU/mL), median (IQR)	1.3 (0.7–2.3)	1.4 (0.8–2.6)	1.2 (0.6–1.9)	0.207 ^+^
Free triiodothyronine (pg/mL), mean (SD)	1.9 (0.5)	1.9 (0.4)	2.2 (0.6)	0.004 ^#^
Free thyroxine (ng/dL), mean (SD)	1.0 (0.3)	1.0 (0.3)	1.0 (0.3)	0.596 ^#^
Partial thromboplastin time (sec), mean (SD)	28.0 (6.7)	28.6 (7.1)	27.2 (5.9)	0.070 ^#^
Prothrombin time (sec), mean (SD)	13.7 (4.2)	14.2 (5.1)	13.1 (2.3)	0.013 ^#^
Thrombin time (sec), mean (SD)	18.7 (4.7)	18.9 (5.6)	18.4 (3.0)	0.297 ^#^

Data are presented as mean (standard deviation) or median (interquartile range). ^#^ Student’s *t*-test, ^+^ Mann–Whitney U test. **Abbreviations:** mm—millimeter; g—gram; dL—deciliter; mg—milligram; mL—milliliter; mmol—millimole; L—liter; U—unit; uIU—micro international unit; pg—picogram; ng—nanogram; SD—standard deviation; IQR—interquartile range; sec—second; DRP—drug-related problem.

**Table 5:** Prescribed medication use among the study participants according to the ATC system.

Characteristics	All (n = 357)	DRPs > 1 (n = 205)	DRP ≤1 (n = 152)	*p*-Value
		N (%)	N (%)	
Total number of prescribed medications	3136 (100)	2241 (100)	895 (100)	
Number of prescribed medications per person, median (IQR) ^a^	9 (IQR 5–12)	11 (IQR 8–13)	5 (IQR 3–8)	<0.001 ^+^
Polypharmacy	290 (81.2)	199 (97.1)	91 (59.9)	<0.001 *
Exposure to at least 1 ADE	27 (7.6)	24 (11.7)	3 (2.0)	0.001 *
Exposure to at least 1 PIM	242 (67.8)	201 (98.0)	41 (27.0)	<0.001 *
Exposure to at least 1 DDI	256 (71.7)	202 (98.5)	54 (35.5)	<0.001 *
**Prescribed medications according to ATC classes and codes**				
A02 Drugs for acid-related disorders	151 (42.3)	115 (56.1)	36 (23.7)	<0.001 *
A03 Drugs for functional gastrointestinal disorders	28 (7.8)	20 (9.8)	8 (5.3)	0.118 *
A06 Drugs for constipation	78 (21.8)	50 (24.4)	28 (18.4)	0.177 *
A10 Drug used in diabetes	100 (28.0)	74 (36.1)	26 (17.1)	<0.001 *
A11 Vitamins	194 (54.3)	129 (62.9)	65 (42.8)	<0.001 *
A12 Mineral supplements	139 (38.9)	98 (47.8)	41 (27.0)	<0.001 *
B01 Antithrombotic agents	165 (46.2)	123 (60.0)	42 (27.6)	<0.001 *
B05 Blood substitutes	60 (16.8)	42 (20.5)	18 (11.8)	0.031 *
C01 Cardiac therapy	76 (18.8)	60 (29.3)	7 (4.6)	<0.001 *
C02 Antihypertensives	63 (17.6)	52 (25.4)	11 (7.2)	<0.001 *
C03 Diuretics	66 (18.5)	59 (28.8)	7 (4.6)	<0.001 *
C07 Beta blocking agents	137 (38.4)	98 (47.8)	39 (25.7)	<0.001 *
C08 Calcium channel blockers	158 (44.3)	104 (50.7)	54 (35.5)	0.004 *
C09 Agents acting on the renin-angiotensin system	89 (24.9)	54 (26.3)	35 (23.0)	0.474 *
C10 Lipid modifying agents	222 (62.2)	148 (72.2)	74 (48.7)	<0.001 *
D Dermatologicals	13 (3.6)	8 (3.9)	5 (3.3)	0.760 *
H02 Corticosteroids	53 (14.8)	31 (15.1)	22 (14.5)	0.865 *

Data are presented as n (%) or median (interquartile range) * Chi-square test, ^+^ Mann–Whitney U test. ^a^ Variable represented as median (IQR). **Abbreviations:** IQR—interquartile range; ATC—Anatomical Therapeutic Chemical; DRP—drug-related problem; PIM—potentially inappropriate medication; DDI—drug–drug interaction; ADE—adverse drug event.

**Table 6:** Prescribed medication use among the study participants according to the ATC system.

Characteristics	All (n= 357)	DRPs > 1 (n = 205)	DRP ≤ 1 (n = 152)	*p*-Value
		N (%)	N (%)	
**Prescribed medications according to ATC classes and codes**				
H Systemic hormonal preparations	51 (14.3)	37 (18.0)	14 (9.2)	0.018 *
H02 Corticosteroids	53 (14.8)	31 (15.1)	22 (14.5)	0.865 *
H03 Thyroid therapy	24 (6.7)	18 (8.8)	6 (3.9)	0.071 *
J01 Antibacterial drugs	111 (31.1)	62 (30.2)	49 (32.2)	0.687 *
J05 Antivirals for systemic use	57 (16.0)	34 (16.6)	23 (15.1)	0.711 *
L01 Antineoplastic agents	8 (2.2)	4 (2.0)	4 (2.6)	0.727 *
L03 Immunostimulants	7 (2.0)	7 (3.4)	0 (0.0)	0.022 *
L04 Immunosuppressants	40 (11.2)	29 (14.1)	11 (7.2)	0.041 *
M05 Drugs for treatment of bone diseases	6 (1.7)	6 (2.9)	0 (0.0)	0.040 *
N02 Analgesics	101 (28.3)	64 (31.2)	37 (24.3)	0.154 *
N03 Antiepileptics	54 (15.1)	42 (20.5)	12 (7.9)	0.001 *
N05 Psycholeptics	60 (16.8)	52 (25.4)	8 (5.3)	<0.001 *
N05A Antipsychotics	14 (3.9)	14 (6.8)	0 (0.0)	0.001 *
N05B-N05C Anxiolytics, sedatives and hypnotics	48 (13.4)	40 (19.5)	8 (5.3)	<0.001 *
N06 Psychoanaleptics	21 (5.9)	19 (9.3)	2 (1.3)	0.002 *
N06A Antidepressants	22 (6.2)	18 (8.8)	4 (2.6)	0.017 *
N06D Anti-dementia drugs	2 (0.6)	2 (1.0)	0 (0.0)	0.510 *
R03 Drugs for obstructive airway diseases	53 (14.8)	36 (17.6)	17 (11.2)	0.094 *
R05 Cough and cold preparations	38 (10.6)	29 (14.1)	9 (5.9)	0.013 *
R06 Antihistamines for systemic use	45 (12.6)	32 (15.6)	13 (8.6)	0.047 *

Data are presented as n (%). * Chi-square test. **Abbreviations:** ATC—Anatomical Therapeutic Chemical; DRP—drug-related problem.

**Table 7:** Prevalence of each type of drug-related problem in the study population.

Characteristics	N (%)
**Number of participants with the different combinations of criteria of PIM, DDI, ADE**	357 (100)
None	54 (15.1)
Only PIM	41 (11.5)
Only DDI	54 (15.1)
Only ADE	3 (0.8)
ADE + PIM	3 (0.8)
ADE + DDI	4 (1.1)
PIM + DDI	181 (50.7)
ADE + PIM + DDI	17 (4.8)
**Potentially inappropriate medications (PIMs), older person**	357 (100)
No PIM	115 (32.2)
At least 1 PIMs	242 (67.8)
1	80 (22.4)
2	58 (16.2)
≥3	104 (29.1)
**Drug–drug interactions (DDIs), older person**	357 (100)
Without DDI	101 (28.3)
At least 1 DDI	256 (71.7)
With 1–5 DDIs	171 (47.9)
With 6–10 DDIs	67 (18.8)
With > 10 DDIs	18 (5.0)
**Severity of adverse drug events (ADEs)**	27 (100)
Grade 1 Mild	0 (0)
Grade 2 Moderate	0 (0)
Grade 3 Severe	15 (55.6)
Grade 4 Life-threatening consequences	12 (44.4)
Grade 5 Death related to ADE	0 (0)

Data are presented as n (%). **Abbreviations:** ADE—adverse drug event; PIM—potentially inappropriate medication; DDI—drug–drug interaction.

**Table 8:** Multivariate analysis of factors associated with exposure to > 1 criterion of DRPs.

The Associated Factors	Multivariate ^a^	*p*-Value
	OR (95%CI)	
**Baseline and clinical characteristics**		
History of previous fall in the past 12 months	1.79 (0.89–3.58)	0.097
Age > 70 years	1.09 (0.59–2.04)	0.776
Female	1.44 (0.79–2.62)	0.235
Barthel ADL score < 12	0.48 (0.16–1.44)	0.192
Lawton IADL score < 8	1.24 (0.62–2.51)	0.552
MoCA score < 25	0.91 (0.43–1.93)	0.805
CCI score ≥ 5	1.22 (0.59–2.50)	0.583
CFS score ≥ 5	0.95 (0.43–2.11)	0.907
**Medication profiles**		
Polypharmacy	8.25 (3.14–21.70)	<0.001 *
A02 Drugs for acid-related disorders	3.82 (2.08–7.00)	<0.001 *
A10 Drug used in diabetes	2.33 (1.19–4.59)	<0.001 *
C01 Cardiac therapy	3.19 (1.29–7.89)	0.012
C03 Diuretics	8.20 (3.11–21.65)	<0.001 *
N05 Psycholeptics	4.59 (1.80–11.69)	0.001 *
N06 Psychoanaleptics	5.97 (1.21–29.34)	0.028

* *p* < 0.01. ^a^ Adjusted for age, sex, Barthel ADL score, Lawton IADL score, MoCA score, CCI score, CFS score, a history of previous falls in the past 12 months and significant variables from the unadjusted model. Data are presented as odds ratio (95% confidence interval). **Abbreviations:** DRP—drug-related problems; OR—odds ratio; CI—confidence interval.

## Data Availability

The data presented in the current study are not publicly available due to ethical restrictions. However, data are available from the corresponding author upon reasonable request.

## Supplementary Materials

The following supporting information can be downloaded at: https://www.mdpi.com/article/10.3390/jcm13061638/s1. Table S1: potentially inappropriate medications in the study population according to the 2019 Beer Criteria; Table S2: lists of the most common drug interactions with their potential effects, according to the Micromedex Drug Interaction Database; Table S3: severity of adverse drug events; Table S4: category of adverse drug events; Table S5: category of medications associated with adverse drug events; Table S6: preventability of adverse drug events.
