# Supplementary material for: Drug Related Problems among Older Inpatients at a Tertiary Care Setting
Source: J Clin Med. 2024 Mar 13;13(6):1638. doi: 10.3390/jcm13061638 (PMC10971276; doi:10.3390/jcm13061638)
Supplement: Supplementary file 1 [file jcm-13-01638-s001.zip › Table S1. Potentially inappropriate medications of the population study according to the 2019 Beer Criteria_JCM.pdf]

Table S1. Potentially inappropriate medications of the population study according to the 2019 Beer Criteria

Category of PIMs	PIMs (n=612) N (%)
**PIMs following Beer criteria 2019**	612 (100)
**PIMs Independence of Medical Condition**	324 (52.9)
Proton pump inhibitors	106 (17.3)
Benzodiazepines	59 (9.6)
Anticholinergics	59 (9.6)
**PIMs due to Drug-Disease or Drug-Syndrome Interactions**	70 (11.4)
Benzodiazepines	26 (4.2)
Antiepileptics	13 (2.1)
Opioids	12 (1.9)
**Drugs to be used with caution in older adults**	183 (29.9)
Diuretics	70 (11.4)
Trimethoprim-sulfamethoxazole	22 (3.6)
Antipsychotics	13 (2.1)
**PIMs due to drug-drug interactions**	8 (1.3)
Amitriptyline-Orphenadrine	2 (0.3)
Amiodarone-Warfarin	1 (0.2)
Orphenadrine-Cetirizine	1 (0.2)
**Drugs to be adjusted in older adults with varying levels of kidney function**	27 (4.4)
Gabapentin	6 (0.9)
Pregabalin	5 (0.8)
Bactrim	4 (0.6)

Data are presented as n (%)

Abbreviations: PIM, potentially inappropriate medication.
