# Supplementary material for: Drug Related Problems among Older Inpatients at a Tertiary Care Setting
Source: J Clin Med. 2024 Mar 13;13(6):1638. doi: 10.3390/jcm13061638 (PMC10971276; doi:10.3390/jcm13061638)
Supplement: Supplementary file 1 [file jcm-13-01638-s001.zip › Table S2. Lists of most common drug interactions with potential effects according to Micromedex Drug Interaction Database_JCM.pdf]

Table S2. Lists of most common drug interactions with potential effects according to Micromedex Drug Interaction Database

Drug combination	Potential effects	(n=1210) N (%)
**Major drug interactions**		
Amlodipine + Simvastatin	Increased risk of myopathy and rhabdomyolysis	36 (2.9)
Aspirin + Clopidogrel	Increased risk of bleeding	26 (2.1)
Clopidogrel + Omeprazole	Reduced plasma concentrations of clopidogrel active metabolite and reduced antiplatelet activity	24 (1.9)
Aspirin + Furosemide	Decreased diuretic effectiveness and increased nephrotoxicity	16 (1.3)
Aspirin + Enoxaparin	Increased risk of bleeding	9 (0.7)
**Moderate drug interactions**		
Atorvastatin + Clopidogrel	Decreased formation of clopidogrel active metabolite	34 (2.8)
Aspirin + Calcium	Decreased salicylate effectiveness	22 (1.8)
Aspirin + Carvedilol	Increased blood pressure	17 (1.4)
Ferrous + Omeprazole	Reduced non-heme iron bioavailability	17 (1.4)
Aspirin + Insulin	Increased risk of hypoglycemia	16 (1.3)
**Minor drug interactions**		
Calcium carbonate + Ferrous fumarate	Decreased iron effectiveness	26 (2.1)
Ferrous fumarate + Sodium bicarbonate	Decreased iron effectiveness	19 (1.6)
Furosemide + Hydralazine	Enhanced diuretic response to furosemide	12 (0.9)
Cyanocobalamin + Omeprazole	Decreased cyanocobalamin absorption	11 (0.9)

Data are presented as n (%)
