# Supplementary material for: Drug Related Problems among Older Inpatients at a Tertiary Care Setting
Source: J Clin Med. 2024 Mar 13;13(6):1638. doi: 10.3390/jcm13061638 (PMC10971276; doi:10.3390/jcm13061638)
Supplement: Supplementary file 1 [file jcm-13-01638-s001.zip › Table S3. Severity of adverse drug events_JCM.pdf]

Table S3. Severity of adverse drug events

Category of ADEs' severity	ADE (n=27) N (%)
Grade 1 Mild	0 (0)
Grade 2 Moderate	0 (0)
Grade 3 Severe	15 (55.6)
Grade 4 Life-threatening consequences	12 (44.4)
Grade 5 Death related to ADE	0 (0)
**Total ADEs**	**27 (100)**

Data are presented as n (%)

Abbreviations: ADE, adverse drug event
