# Supplementary material for: Drug Related Problems among Older Inpatients at a Tertiary Care Setting
Source: J Clin Med. 2024 Mar 13;13(6):1638. doi: 10.3390/jcm13061638 (PMC10971276; doi:10.3390/jcm13061638)
Supplement: Supplementary file 1 [file jcm-13-01638-s001.zip › Table S4. Category of adverse drug events_JCM.pdf]

Table S4. Category of adverse drug events

Types of ADEs	ADE (n=27)<br>N (%)
**Neurological problems**	2 (7.4)
Alteration of consciousness	1 (3.7)
Myopathy	1 (3.7)
**Skin problems**	1 (3.7)
Anaphylactic shock with rash	1 (3.7)
**Respiratory problems**	2 (7.4)
Pneumonitis	1 (3.7)
Diffuse interstitial lung disease	1 (3.7)
**Cardiovascular problems**	2 (7.4)
Pericarditis	1 (3.7)
Congestive heart failure	1 (3.7)
**Renal problems**	1 (3.7)
Lactic acidosis with acute kidney injury	1 (3.7)
**Hematological problems**	19 (70.4)
Pancytopenia	3 (11.1)
Agranulocytosis	3 (11.1)
Intraabdominal bleeding	1 (3.7)
Febrile neutropenia	7 (25.9)
Hematuria	1 (3.7)
Upper gastrointestinal bleeding	3 (11.1)
Lower gastrointestinal bleeding	1 (3.7)

Data are presented as n (%)

Abbreviations: ADE, adverse drug event
