# Supplementary material for: Drug Related Problems among Older Inpatients at a Tertiary Care Setting
Source: J Clin Med. 2024 Mar 13;13(6):1638. doi: 10.3390/jcm13061638 (PMC10971276; doi:10.3390/jcm13061638)
Supplement: Supplementary file 1 [file jcm-13-01638-s001.zip › Table S5. Category of medications associated with adverse drug events_JCM.pdf]

Table S5. Category of medications associated with adverse drug events

Category of medications associated with ADEs	ADEs (n=27) N (%)
A10 Drug used in diabetes	1 (3.7)
A11 Vitamins	1 (3.7)
B01 Antithrombotic agents	6 (22.2)
C02 Antihypertensives	1 (3.7)
C10 Lipid modifying agents	1 (3.7)
J01 Antibacterial drugs	1 (3.7)
J05 Antivirals for systemic use	1 (3.7)
L01 Antineoplastic agents	13 (48.1)
L04 Immunosuppressants	1 (3.7)
M04 Antigout preparations	1 (3.7)

Data are presented as n (%)

Abbreviations: ADE, adverse drug event
