# Supplementary material for: Drug Related Problems among Older Inpatients at a Tertiary Care Setting
Source: J Clin Med. 2024 Mar 13;13(6):1638. doi: 10.3390/jcm13061638 (PMC10971276; doi:10.3390/jcm13061638)
Supplement: Supplementary file 1 [file jcm-13-01638-s001.zip › Table S6. Preventability of adverse drug events_JCM.pdf]

Table S6. Preventability of adverse drug events

Category of preventability	ADEs (n=27)<br>N (%)
**Preventable ADEs**	10 (37.0)
LGIB from warfarin	1 (3.7)
UGIB from enoxaparin	2 (7.4)
UGIB from dabigatran	1 (3.7)
Hematuria from enoxaparin	1 (3.7)
Intraabdominal bleeding from warfarin	1 (3.7)
Myopathy from colchicine	1 (3.7)
Lactic acidosis and acute kidney injury from metformin	1 (3.7)
Alteration of consciousness from vitamin D	1 (3.7)
Neutropenia from methotrexate	1 (3.7)

Data are presented as n (%)

Abbreviations: ADE, adverse drug event; LGIB, lower gastrointestinal bleeding; UGIB, upper gastrointestinal bleeding
